# Mitofusin-2 mediates doxorubicin sensitivity and acute resistance in Jurkat leukemia cells

**DOI:** 10.1016/j.bbrep.2020.100824

**Published:** 2020-10-08

**Authors:** Carl W. Decker, Jerome Garcia, Kristelle Gatchalian, Deronisha Arceneaux, Clarice Choi, Derick Han, Jeniffer B. Hernandez

**Affiliations:** a Keck Graduate Institute, Henry E. Riggs School of Applied Life Sciences, 535 Watson Drive, Claremont, CA, 91711, USA; b Keck Graduate Institute, Department of Pharmaceutical Sciences, School of Pharmacy and Health Sciences, 535 Watson Drive, Claremont, CA, 91711, USA; c University of La Verne, Department of Biology, 1950 3rd Street, La Verne, CA, 91750, USA; d Pitzer College, 1050 N Mills Ave, Claremont, CA, 91711, USA

**Keywords:** Mitochondrial fusion, Doxorubicin, MFN-2, Sensitivity, OXPHOS

## Abstract

Mitochondria oscillate along a morphological continuum from fragmented individual units to hyperfused tubular networks. Their position at the junction of catabolic and anabolic metabolism couples this morphological plasticity, called mitochondrial dynamics, to larger cellular metabolic programs, which in turn implicate mitochondria in a number of disease states. In many cancers, fragmented mitochondria engage the cell with the biosynthetic capacity of aerobic glycolysis in service of proliferation and progression. Chemo-resistant cancers, however, favor remodeling dynamics that yield fused mitochondrial assemblies utilizing oxidative phosphorylation (OXPHOS) through the electron transport chain (ETC). In this study, expression of Mitofusin-2 (MFN-2), a GTPase protein mediator of mitochondrial fusion, was found to closely correlate to Jurkat leukemia cell survival post doxorubicin (DxR) assault. Moreover, this was accompanied by dramatically increased expression of OXPHOS respiratory complexes and ATP Synthase, as well as a commensurate escalation of state III respiration and respiratory control ratio (RCR). Importantly, CRISPR knockout of MFN-2 resulted in a considerable decrease of doxorubicin (DxR) median lethal dose compared to a treated wildtype control, suggesting an important role of mitochondrial fusion in chemotherapy sensitivity and acute resistance.

## Highlights

• Doxorubicin induces mitochondrial fusion in surviving jurkat cells
• Fused mitochondria in surviving cells increase respiration and mitochondria coupling
• Mitofusin-2 knockout sensitizes cells to doxorubicin

## Graphical abstract

**Figure:**
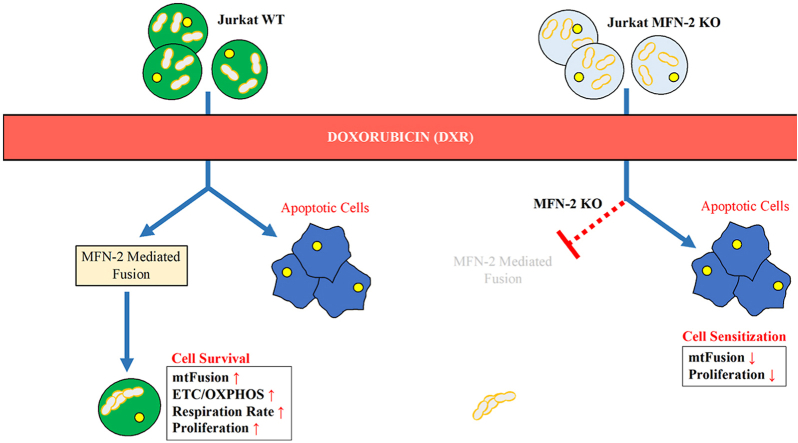

## 1 Introduction

T-cell leukemias are a group of blood cancers that are among the most common in the world, affecting over 2.3 million people globally and causing 353,000 deaths annually [1,2]. Notably, leukemia is the most common pediatric cancer [3]. Standard of care treatments routinely incorporate the CHOP (cyclophosphamide, hydroxydaunorubicin, oncovin, prednisone) chemotherapy regimen [4], which includes the intercalating anthracycline doxorubicin (DxR). While useful in treating early-to mid-stage lymphomas [5], sustained DxR-inclusive CHOP also presents significant side effects to patients, including dilated cardiomyopathy, congestive heart failure, and death. Moreover, cancerous cells exposed to repeated dosing of chemotherapy regimens, as well as those that are recurrent, are frequently less primed to undergo mitochondria-mediated intrinsic apoptosis [6], making them less sensitive to additional applications of chemotherapy. This is significant, as lymphoblastic leukemias have a relapse rate of 10–25% in children [7,8], for which prognoses are dramatically impacted [9]. As such, mitochondrial action in leukemia cells under chemotherapeutic assault serves as a potentially crucial area in which to research drug sensitivity and efficacy.

Mitochondria are active, double-membrane bound hubs of bioenergetics, cell signaling, and redox balance that fluctuate along a spectrum of fused superstructures to smaller, fragmented organelles. Importantly, these morphological changes-together called mitochondrial dynamics-are closely connected to larger metabolic schemes. Specifically, fission has been shown to favor glycolysis [10], while several studies have linked fusion with induction of oxidative phosphorylation/OXPHOS [[11], [12], [13], [14]], and are mediated by the action of a family of intracellular GTPase proteins. For example, the GTPase dynamin related protein (DRP-1), when phosphorylated at serine_616_, localizes with Mitochondrial Fission Factor (Mff) at the outer mitochondrial membrane (OMM), dividing targeted mitochondria at scission sites to induce a fragmented network morphology and a switch to glycolytic metabolism. Conversely, the mitofusin proteins (MFN-1,2) function to merge the mitochondrial outer membrane (MOM) of fusing mitochondria. The relative balance of these proteins and their subsequent action are essential to normal cell physiology, and their dysregulation has been connected to a number of cancers [15]. Conversely, mitochondrial fusion has been shown to inhibit migration in breast cancer, establishing a linking mechanism between cancer invasiveness and mitochondrial dynamics [16]. Unfortunately, many similar links between mitochondrial dynamics and cell sensitivity to chemotherapy remain unexplored or poorly characterized.

The present study aimed to address this dearth by examining the effects of doxorubicin on the mitochondrial dynamics in leukemia Jurkat cells, with particular attention paid to mitochondrial phenotype and respiratory capacity in cells surviving chemotherapeutic assault. We also sought to characterize the relationship between those mitochondrial dynamics and cellular sensitivity to DxR. Our subsequent data show that both MFN-2 and OXPHOS respiratory complexes are significantly upregulated in surviving leukemia cells, and that MFN-2 knockout substantially increases Jurkat sensitivity to DxR, providing a potential target for increasing chemotherapy efficacy and reduction of side effects.

## 2 Materials and methods

### 2.1 Cell culture and chemotherapy application

Jurkat peripheral blood t-lymphocytes from ATCC (TIB-152™) were grown in RPMI medium supplemented with 2 mm glutamine, 10% fetal bovine serum, and penicillin/streptomycin (50 μM mL^−1^), all from ThermoFisher/Gibco (Waltham, Massachusetts), in a humidified atmosphere containing 5% CO_2_/95% air at 37 °C.

### 2.2 Cell viability

To separate live cells, suspension was spun down at 118×*g* on an Eppendorf 5702R centrifuge with a 13.2 cm radius (Eppendorf rotor A-4-38). For trypan exclusion, a 0.4% trypan solution (SigmaAldrich, St. Louis, Missouri) was applied to fresh cell suspension in a 1:1 ratio. Flow cytometry was performed per the protocol in prior studies [17].

### 2.3 Mfn-2 knockout CRISPR vector transfection

Mfn2 CRISPR/Cas9 KO Plasmid was obtained from Santa Cruz Biotechnology (Santa Cruz, California), mixed in proportion with SCBT UltraCruz Transfection Reagent (Santa Cruz, California) and OPTI-MEM Reduced Serum Media (ThermoFisher, Walthham, Massachusetts), and applied to cells in-vitro for 72 h, mixing suspension every 12 h.

### 2.4 Immunoblotting

Western blot was conducted as per our previous work [18]. Aliquots of cytoplasmic or mitochondrial extracts were fractionated by electrophoresis on 8–12% SDS polyacrylamide gels (BioRad, Hercules, CA). Subsequently, proteins were transferred to PVDF membranes and blots were blocked with 1% casein (w/v) nonfat milk dissolved in Tris-buffered saline (TBS) with Tween-20 (BioRad, Hercules, CA). Total OXPHOS cocktail, DRP-1, pDRP-1, and MFN-2 primary antibodies were obtained from Abcam (Cambridge, UK). . All blots shown are representative samples from 3 to 7 experiments.

### 2.5 Real-time quantitative PCR

RT-qPCR was performed via our previous work [19]***.*** Total RNA was extracted from treated cells through liquid phase separation via use of TriPure reagent (Roche Diagnostics, Indianapolis, IND, USA), chloroform, and 2 Propanol. The resulting RNA was resuspended in RNase-free water and quantified using NanoQuant Infinite 200 Pro. With 1 μg of total RNA, a cDNA library was synthesized using the AzuraQuant cDNA Synthesis Kit (Azura Genomics, Massachusetts, USA) and MJ Research PTC 200 Gradient Thermal Cycler; cDNA was subsequently diluted 1:10. For the qPCR, approximately 100 ng of cDNA template per treatment condition was mixed with 1× AzuraQuant Green Fast qPCR Mix HiRox (Azura Genomics, Massachusetts, USA), 400 nM Forward Primers (MFN2 and DRP1), 400 nM Reverse Primers (MFN2 and DRP1), and RNase free water. 18S was also amplified as an internal control. Samples were then loaded into a 96 well plate at 20 μL total volume per well with each condition run as triplicates. A Roche Lightcycler 96 was set for 1 cycle at 95 °C for 2 min, two-step amplification for 1 Cycle at 95 °C for 5 s then 40 cycles of 60 °C for 25 s, and 1 cycle at 37 °C for 30 s. The resulting Cq data was normalized with the 18S control to limit variability. The data was then analyzed using the 2^−ΔΔCT^ method. All data was quantified using the Roche Lightcycler 2.0 software.

### 2.6 Mitochondrial respiration measurements

Respiration was measured in freshly isolated mitochondria according to our prior work [20] by monitoring oxygen consumption with a Clark‐type electrode (Hanstech, UK) in respiration buffer containing 230 mm mannitol, 70 mm sucrose, 30 mm Tris–HCl, 5 mm KH_2_PO_4_, 1 mm EDTA, pH 7.4 [9]. Control and treated Jurkat suspensions with digitonin applied were added to 1 mL of respiration buffer and oxygen consumption monitored in the presence of mitochondrial Complex I substrates (glutamate/malate 7.5 mM) with or without ADP (250 μM). State IV respiration is defined as respiration in the presence of substrate, while state III respiration in is defined as respiration in the presence of both substrates plus ADP. The RCR is defined as state III/state IV.

### 2.7 Statistical analysis

Statistical analyses were performed using the Student's t-test for unpaired data and ANNOVA for multiple sets. P ≤ 0.05 was defined as statistically significant.

## 3 Results

### 3.1 DxR cytotoxicity

**Fig. 1:**
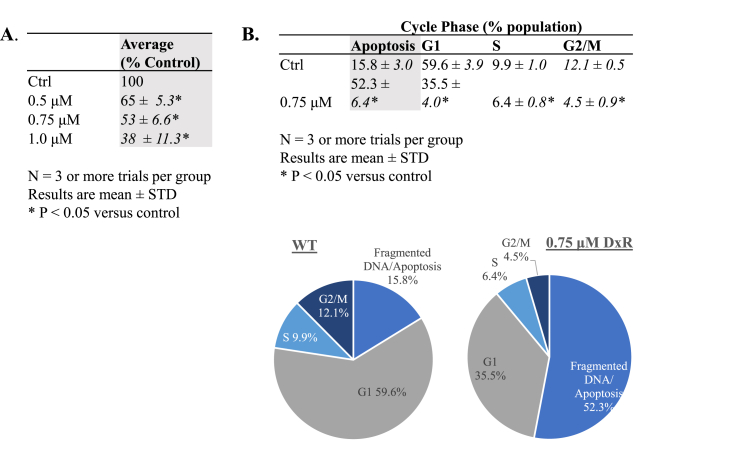
**A)** Doxorubicin (DxR) cytotoxicity for suspended jukrat t-cell cultures over a treatment interval of 24 h determined through trypan exclusion. Viable population is reduced dose-dependently with increases to DxR treatment concentration, with approximate acute median lethal dose (LD_50_) achieved at 0.75 μM (53% of untreated control, ± 6.6%). **B)** Cell cycle phase analysis via flow cytometry. Fragmented DNA is used as indication of apoptosis, given DNA intercalation mechanism of DxR. DxR treatment at 0.75 μM increases DNA fragmented cell proportion from 15.8% ± 3.0% of population to 52.3% ± 6.4% versus untreated WT (wildtype). Additionally, the proportion of proliferative G2/M phase in treated cells is 4.5% ± 0.9, down from 12.1% ± 0.5% in control. Data represent the mean ± SD, n > 3.

Titration of 24-h DxR treatments established the median lethal dose for a Jurkat t-cell culture. A trypan exclusion assay and statistical analysis showed cytotoxicity in a dose-dependent relationship. 0.75 μM DxR was found to reduce cell viability by approximately half (53% of control, ± 6.6), with 0.5 μM and 1.0 μM respectively constituting low (65% of control, ± 5.3 reduction) and high (38% of control, ± 11.3 reduction) dosing states (Fig. 1A). For validation, cell cycle analysis using 0.75 μM DxR corroborated its median dose cytotoxicity, increasing the DNA fragmentation subpopulation (indicative of apoptosis) of treated Jurkats to 52.3% ± 6.4, while reducing the proportion of cells in the proliferative G_2_/M phase to 4.5% ± 0.9, down from 12.1% ± 0.5 in the untreated control (Fig. 1B).

### 3.2 DxR induces changes to mitochondrial remodeling, including MFN-2 mediated mitochondrial fusion

**Fig. 2:**
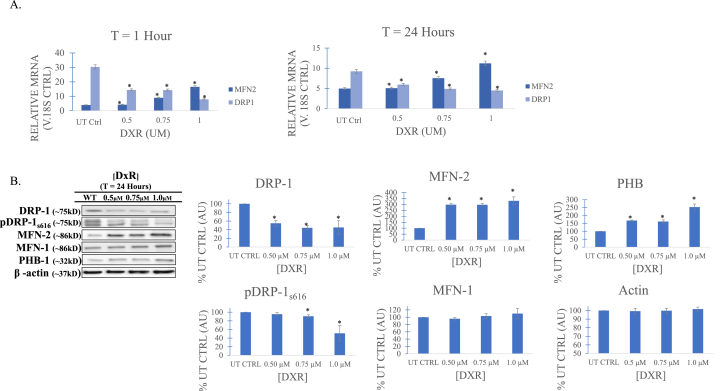
**A)** Real-time quantitative PCR gene detection comparison measuring protein mediators of mitochondrial morphology across doxorubicin (DxR) treatment concentrations and untreated control (UT) at 1 and 24 h relative to an 18s control gene. At 1 h post-DxR treatment, transcript of dynamin related protein 1 (DRP-1), a GTPase mediator of mitochondrial fission, is shown to decrease. This is accompanied by a concurrent increase of MFN-2, a mitochondrial outer membrane fusion mediator, indicating hasty mitochondrial fusion in response to DxR; these same dynamics are observed at a 24 h endpoint. **B)** Immunoblot and densitometry (relative arbitrary units, AU) of mitochondrial dynamics mediators in DxR treated t-cells at 24 h. Corroborating RT-qPCR data, both DRP-1 and activated pDRP-1_s616_ are decreased in response to treatment. MFN-2 is likewise shown to increase as in RT-qPCR assay, though an increase of MFN-1 was determined to be statistically insignificant. Prohibitin 1 (PHB-1), a pleiotropic scaffolding protein known to stabilize mitochondrial inner membrane proteins and support cristae morphogenesis and maintenance, is upregulated in treated conditions compared to control. Taken together, data suggest MFN-2 mediated fusion in response to DxR, beginning at the latest 1 h after treatment, through to a 24 h interval. Data represent the mean ± SD, n > 3.

Versus control, DxR treatment across low (0.25 μM), medium (0.50 μM), and high (1.0 μM) concentrations induced rapid transcription of fusion mediating MFN-2 beginning at 1hr and continuing through 24-h (Fig. 2B). Western immunoblot revealed expression of MFN-2 was greatly increased for all treated groups versus control. Interestingly, protein expression of Mitofusin-1 (MFN-1), a homolog of MFN-2, only marginally increased. The pleiotropic protein Prohibtin-1 (PHB-1), which forms ring structures at the mitochondria inner membrane to support fusion morphology and OXPHOS, increased with DxR concentration, suggesting inner membrane fusion maintenance. Conversely, production of DRP-1, both in its total pool and activated phosphorylated (serine_616_) forms, was found to be inversely correlated to DxR concentration beginning at the 1-h timepoint (Fig. 2B), indicating suppression of mitochondrial fission.

### 3.3 Oxidative phosphorylation, respiration rate, and mitochondrial coupling are upregulated and enhanced in cells that survive DxR assault

**Fig. 3:**
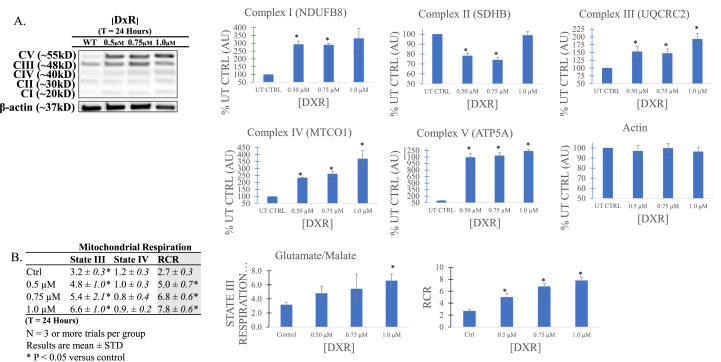
**A)** Immunoblot of electron transport chain respiratory proteins. Compared to untreated control (UT), complexes I, III, and IV are greatly upregulated, with the largest increase in expression occurring in complex V (ATP Synthase), indicating increased ATP production via oxidative phosphorylation. Interestingly, complex II (succinate dehydrogenase) is not increased, perhaps to further produce ATP through glutamate/malate-driven respiration via complex I, which is more efficient per molecule of glucose. Data are representative of n > 3. **B)** Oxygen consumption/mitochondrial respiration rates (states III and IV) and RCR measured using polarographic oxygen electrode. State III respiration, in which ADP and substrate (for complex I, glutamate) are saturated, is indicative of maximal electron transport chain (ETC) respiration. The increased expression of oxidative phosphorylation (OXPHOS) proteins observed in DxR treated jurkats is accompanied by a commensurate increase in glutamate/malate-driven state III respiration. State IV respiration, or minimal respiratory capacity, is only marginally altered, suggesting very little proton leak or mitochondrial damage. Respiratory control ratio (RCR), a measure of ATP production efficiency via mitochondrial coupling, is increased with doxorubicin (DxR) treatment concentration in a dose dependent manner. Data represent the mean ± SD, n > 3.

Mitochondrial fusion is associated with energy production via electron transport and oxidative phosphorylation (OXPHOS). To assess if our observation of upregulated MFN-2, along with its mediated fusion, predicated subsequent increases in OXPHOS activity, we probed for subunits of each respiratory chain complex, I–V (Fig. 3A). Significant increases were observed for complexes I, III, and IV, with a substantial 10-fold surge occurring for complex V, ATP Synthase. Interestingly, expression for complex II, succinate dehydrogenase, was slightly decreased compared to control.

To determine if the upregulation of mitochondrial fusion and OXPHOS complexes in treated cells accompanied respiration rate changes, we introduced a glutamate/malate respiratory substrate to facilitate electron entry into the ETC via Complex I. Using ADP + glutamate/malate-generated NADH, which facilitates shuttling electrons to Complex I, we observed that mitochondria in treated cells increase state III respiration compared to control (Fig. 3B). This corresponded with lower state IV respiration (however not significant) and a statistically significant escalation in the respiratory control ratio (RCR; state III/state IV), indicating increased mitochondrial coupling and more efficient ATP production (Fig. 3B).

### 3.4 MFN-2 significantly increases chemoresistance and insensitivity

**Fig. 4:**
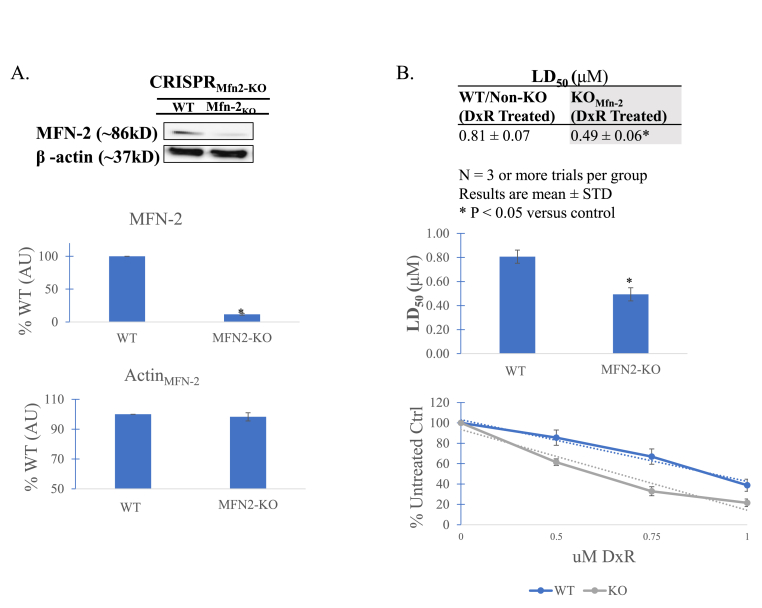
**A)** Validation immunoblot of clustered regularly interspaced short palindromic repeats. CRISPR MFN-2 knockout. Vector was shown to be effective in target gene knockout. Data is representative of n > 3. **B)** New acute median lethal dose established for MFN-2 KO jurkat t-cells. Compared to treated wildtype control (WT), MFN-2 KO cells demonstrate significantly lower LD_50_, decreasing from 0.81 μM to 0.49 μM, respectively. Data suggest that MFN-2 mediated mitochondrial fusion contributes to jurkat t-cell insensitivity and subsequent survival. Data represent the mean ± SD, n > 3.

To deduce the role of MFN-2 in the DxR drug response of Jurkats, we next knocked out its expression through transfection of an MFN-2 KO CRISPR vector. Knockout was validated through western immunoblot (Fig. 4A). To ensure that there was no compensatory MFN-1 expression to induce unaccounted fusion, we probed for it again, finding little change (data not shown). DxR was administered at 0.50 μM, 0.75 μM, and 1.0 μM to both wildtype control and MFN-2 knockout cultures for a duration of 24 h. Compared to control, median lethal dose for MFN-2-KO was reduced from 0.81 μM in WT to 0.49 μM in KO, indicating significant sensitization in MFN-2 deficient cells (Fig. 4B).

## 4 Discussion

DxR is a standard-of-care nonselective anthracycline molecule routinely used in the treatment of leukemia [21]. In this study, we demonstrated that Jurkat leukemia cells, if they are to survive a DxR assault, must quickly change mitochondrial morphology, rates of oxidative phosphorylation, and respiratory capacity. Specifically, we show that mitochondrial fusion mediated by MFN-2 (but not MFN-1), as well as significant increases in expression for particular OXPHOS respiratory proteins and mitochondrial respiration rate, are required for Jurkat cell survival at median lethal DxR doses. Together these data suggest that mitochondrial dynamics, as well as their associated metabolic programs, play an important role in leukemia responses to DxR chemotherapy, and may also account for subsequent DxR chemoresistance and desensitization.

As a DNA intercalator, DxR inhibits the action of topoisomerase II, generating DNA damage and thus preventing biosynthesis and additional cancer growth [22]. Though effective in treating many cancers, its use is limited by several side effects, especially cardiomyopathy [23], which presents a need to maximize its efficacy by reducing its therapeutic dose concentration. At higher concentrations, previous works have linked DxR-induced mitochondrial dysfunction to cardiomyopathy development through decreased respiration of Jurkat cells at 5–10 μM DxR [24]. However, it has been estimated that typical plasma concentrations of DxR in patients is as low as 1–2 μM [25], meaning a gap exists regarding mitochondrial action at smaller clinical concentrations.

Mitochondria possess the capacity to fluctuate between structural morphologies based on cellular stresses, bioenergetic demand [26], and disease state. DRP-1 is activated post-translationally by Cdk1/cyclin B-mediated phosphorylation of Serine-616 [27], translocating to the mitochondrial outer membrane, homo-oligomerizing into a ring structure, and inducing morphological fission [28]. This fission has been observed in several types of cancer [[29], [30], [31]], and studies have found DRP-1 expression to be correlated to mitotic cell cycle phases in which proliferation occurs [32]. In our study, elevated levels of both total and phosphorylated DRP-1 in untreated Jurkats are consistent with this notion, as is the corresponding decreased expression of DRP-1 (Fig. 2) and smaller proportion of G_2_/M phase cells (Fig. 1) in the DxR treated groups. Conversely MFN-2, which induces outer mitochondrial membrane fusion, was observed to increase in treated cells, and while MFN-1 also increased, it was marginal in comparison (Fig. 2). Additionally, prohibitin protein 1 (PHB-1), which assembles at the mitochondria inner membrane to facilitate mitochondrial fusion and stabilize OXPHOS proteins [33], was found to increase with DxR treatment, particularly at 1 μM (Fig. 2). Together, these data suggest a role for mitochondrial dynamics in Jurkat responses to DxR, and in particular, implicate MFN-2 mediated fusion in insensitivity and survival.

Fused mitochondria are associated with oxidative phosphorylation-driven bioenergetics [34], and while healthy differentiated cells typically employ the OXPHOS metabolic program for energy, it is well known that cancer cells are aerobically glycolytic. Although less efficient at ATP generation per molecule of glucose, this transformation from oxidative metabolism to aerobic glycolysis, observed first by Otto Warburg, permits cancer cells to utilize several of the biosynthetic precursors required to support and sustain rapid proliferation [35]. Our data corroborate this process, with a relatively small amount of OXPHOS respiratory chain proteins in the untreated group. Interestingly, after all DxR treatments, expression of OXPHOS proteins (and especially Complex V), were dramatically increased, with complex II providing the lone exception (Fig. 3A). The reasons for this preferential utilization of NADH and the proton pump faculty of Complex I aren't clear, though a possible explanation is that FADH_2_ electron transfer bypasses Complex I [36] to instead feed into Complex II, which doesn't translocate protons and thus is a less efficient energy production method.

We next assessed the relationship between increased OXPHOS respiratory protein expression and respiration rate (Fig. 3B). In the presence of substrate (for Complex I-driven respiration, glutamate/malate) and inorganic phosphate, mitochondria will consume oxygen. Upon adding exogenous ADP to the mitochondria in a reaction chamber, oxygen consumption significantly increases, as its coupled to the conversion of ADP into ATP. Assuming saturation of both substrate and ADP, mitochondrial respiration is limited only by the intrinsic activity of the respiratory chain itself, achieving maximal, or state III, respiration [37]. When ADP levels are exhausted and the ATP:ADP ratio is very high, minimal respiratory capacity, or state IV respiration, can be measured; any oxygen consumption in this state corresponds exclusively to proton leak. Using a Clark-type polarographic oxygen electrode, we found that state III respiration was positively correlated to DxR concentration relative to control, corroborating the increased expression of respiratory complexes previously observed through immunoblot. Taken together, we observe a hastened respiratory rate facilitated by increased respiratory complex expression in response to DxR treatment, contrary to prior studies at higher DxR doses. Moreover, the increased RCR in treated cells indicates more efficient ATP production and tighter mitochondrial coupling, suggesting that rather than damage mitochondria of surviving cells, DxR forces maximum efficiency.

Finally, to determine the impact of observed MFN-2 mediated fusion in surviving cells, as well as the increase in OXPHOS, MFN-2 was knocked out using a CRISPR-MFN2-KO vector, the action of which was validated by immunoblot (Fig. 4A and B). A prior study found that while knockout or silencing of *both* MFN-1 and MFN-2 eliminates mitochondrial fusion, it results in defective cell growth and widespread heterogeneity of mitochondrial membrane potential; greater outcomes were achieved by knocking out only one [38]. Having observed little MFN-1 modulation in response to DxR, MFN-2 was selected to inhibit mitochondrial fusion and OXPHOS, both of which have roles in chemo-resistant cancers [39,40]. Interestingly, when compared to treated wildtype, MFN-2 KO cells demonstrated significant drug sensitization to DxR (Fig. 4B), with a reduced median lethal dose (0.81 μM for treated wildtype, 0.49 μM for treated KO).

Our work here presents MFN-2 and mitochondrial fusion as targets for enhancing doxorubicin efficacy and increasing cell sensitivity to treatment, which with further research may provide opportunities to reduce adverse side effects and improve clinical outcomes. Moreover, our findings further implicate mitochondrial dynamics not only in the bioenergetics of cancer, but in the metabolism of therapies designed to abate its progress.

## Author statement

Carl W. Decker: conceptualized, methodology, validation, formal analysis, investigation visualization and writing-original draft. Jerome Garcia: conceptualization, methodology, resources and writing-review and editing. Kristelle Gatchalian: validation, investigation and formal analysis. Deronisha Arceneaux: investigation and validation. Clarice Choi: investigation and validation. Derick Han: resources and writing-review and editing. Jeniffer B. Hernandez: methodology, resources, writing-review and editing, and supervision.

## Author contributions

CWD designed the study, performed experiments, and wrote the manuscript. JG performed experiments, analyzed data, and revised the manuscript. KG and CC conducted qPCR experiments. DH revised the manuscript. JH designed the study, analyzed data, revised the manuscript, developed research plan.

## Funding

This research did not receive any specific grant from funding agencies in the public, commercial, or not-for-profit sectors.

## Declaration of competing interest

The authors declare that they have no known competing financial interests or personal relationships that could have appeared to influence the work reported in this paper.
